# Correction: Schicklin et al. Method to Generate Chlorine Dioxide Gas In Situ for Sterilization of Automated Incubators. *Pathogens* 2024, *13*, 1024

**DOI:** 10.3390/pathogens14040368

**Published:** 2025-04-09

**Authors:** Cédric Schicklin, Georg Rauter, Philippe Claude Cattin, Manuela Eugster, Olivier Braissant

**Affiliations:** 1 Bio-Inspired RObots for MEDicine-Laboratory, Department of Biomedical Engineering, University of Basel, 4123 Allschwil, Switzerland; cedric.schicklin@unibas.ch (C.S.); georg.rauter@unibas.ch (G.R.); manuela.eugster@unibe.ch (M.E.); 2 Center for medical Image Analysis & Navigation, Department of Biomedical Engineering, University of Basel, 4123 Allschwil, Switzerland; philippe.cattin@unibas.ch; 3 Neuro Robotics Group, ARTORG Center, University of Bern, 3008 Bern, Switzerland; 4 Department of Neurosurgery, Inselspital, Bern University Hospital, 3008 Bern, Switzerland; 5 Biological Calorimetry Lab, Department of Biomedical Engineering, University of Basel, 4123 Allschwil, Switzerland

**Error in affiliations**

In the original publication [1], Manuela Eugster’s affiliation is incomplete. The corrected affiliation of Manuela Eugster should appear as follows:

^1^ Bio-Inspired RObots for MEDicine-Laboratory, Department of Biomedical Engineering, University of Basel, 4123 Allschwil, Switzerland
^3^ Neuro Robotics Group, ARTORG Center, University of Bern, 3008 Bern, Switzerland
^4^ Department of Neurosurgery, Inselspital, Bern University Hospital, 3008 Bern, Switzerland

The authors state that the scientific conclusions are unaffected. This correction was approved by the Academic Editor. The original publication has also been updated.
