# Supplementary material for: Method to Generate Chlorine Dioxide Gas In Situ for Sterilization of Automated Incubators
Source: Pathogens. 2024 Nov 20;13(11):1024. doi: 10.3390/pathogens13111024 (PMC11597574; doi:10.3390/pathogens13111024)
Supplement: Supplementary file 1 [file pathogens-13-01024-s001.zip › Supplementary S1 Table S1.pdf]

pathogens

MDPI

Supplementary material

Table S1. B.subtilis spores, S.cerevisiae, T7 bacteriophage colony-forming units (CFU)/ml with SD (when applicable) for different chlorine dioxide gas ($gClO_2$) concentrations. ND: not determined SD: standard deviation

CFU/ml	0 PPM	64 PPM	116 PPM	255 PPM	433 PPM	650 PPM
B.subtilis spores	$(9.5±3.5)x10^5$	$(9.1±2.5)x10^5$	$(5.8±0.7)x10^5$	$(2±1.3)x10^2$	0	ND
S.cerevisiae	$(1.7±0.6)x10^6$	$(1.5±0.8)x10^6$	$(5.2±2.8)x10^5$	$(7.7±0.04)x10^4$	0	0
T7 bacteriophage	$(2.4±0.6)x10^8$	$(6±5)x10^4$	$2x10^4$	$1x10^1$	ND	ND
