# Supplementary figures and images for: Method to Generate Chlorine Dioxide Gas In Situ for Sterilization of Automated Incubators
Source: Pathogens. 2024 Nov 20;13(11):1024. doi: 10.3390/pathogens13111024 (PMC11597574; doi:10.3390/pathogens13111024)

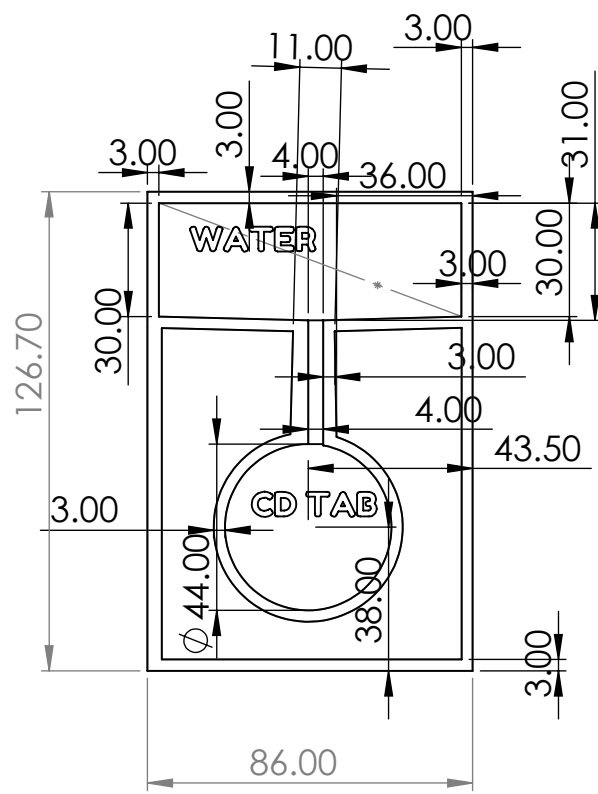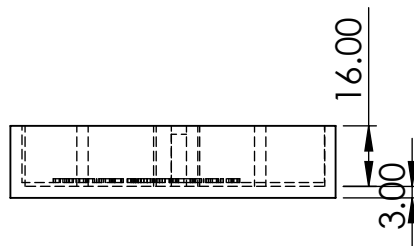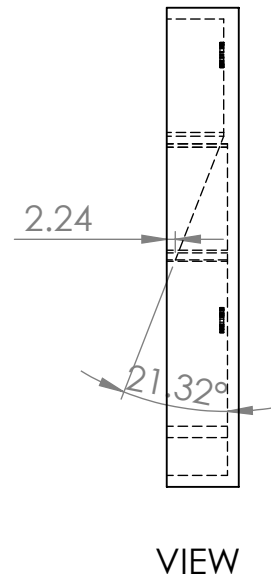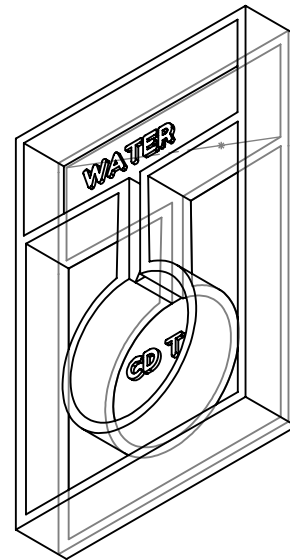

Supplementary material Figure S1: Drawing of the sterilization plate. Dimensions in mm.

Supplement: Supplementary file 1 [file pathogens-13-01024-s001.zip › Supplementary figure S1 MoLiBB_TabletHolder_18ml_v2.pdf]
